# Antioxidants as a Link Between Diabetes and Potential Plant-Based Therapies

**DOI:** 10.3390/antiox15091206

**Published:** 2026-09-20

**Authors:** Alina Kuryłowicz, Leszek Dobrowolski, Monika Słupecka-Ziemilska, Zuzanna Kościów, Marta Kuczeriszka

**Affiliations:** 1 Department of Human Epigenetics, Mossakowski Medical Research Institute, Polish Academy of Sciences, 5 A. Pawińskiego Street, 02-106 Warsaw, Poland; akurylowicz@imdik.pan.pl (A.K.); mslupecka@imdik.pan.pl (M.S.-Z.); 2 Department of Renal and Body Fluid Physiology, Mossakowski Medical Research Institute, Polish Academy of Sciences, 5 A. Pawińskiego Street, 02-106 Warsaw, Poland; lesdobro@imdik.pan.pl; 3 Faculty of Medicine, Łazarski University, 43 Świeradowska Street, 02-662 Warsaw, Poland; zuzia802@gmail.com

**Keywords:** diabetes, diabetes complications, diabetes prevention, plant origin antioxidants, oxidative stress

## Abstract

The pathogenesis and advancement of diabetes mellitus and its associated complications are heavily driven by oxidative stress (OS). It happens when the body produces excessive amounts of reactive oxygen and nitrogen species (RONS). This causes cell damage and leads to chronic health issues, prompting scientific interest in antioxidant-based treatments. In the context of chronic diseases, such as diabetes, the search for new natural bioactive compounds is of particular importance. Within the realm of traditional healthcare, Ayurveda commands significant scientific interest. Botanical materials in the Ayurvedic tradition frequently demonstrate a wide array of physiological benefits, including (among others) hypoglycaemic and antioxidant effects. Because numerous contemporary medications trace their origins back to natural compounds—with metformin serving as a primary example—investigating these traditional botanicals could be instrumental in identifying novel pharmacological treatments. This review focuses on three traditionally used for diabetes: bitter melon (*Momordica charantia*), gurmar (*Gymnema sylvestre*), and jambolan (*Syzygium cumini*). Their therapeutic properties—such as antioxidant, immuno-modulatory, and anti-inflammatory effects—stem from diverse phytochemicals including alkaloids, flavonoids, polypeptides, polysaccharides, saponins and sterols. Together, their overlapping effects create a powerful, unified approach to managing diabetes.

## 1. Introduction

### Diabetes. Diabetes as a Public Health Problem

Diabetes mellitus (DM) represents a severe global public health crisis driven by its rapidly rising prevalence, devastating clinical complications, and immense economic costs [1]. Chronic hyperglycaemia substantially elevates the risk of debilitating conditions such as cardiovascular disease, end-stage renal failure, blindness, and lower-limb amputations, which collectively contribute to millions of premature deaths globally. Beyond the direct clinical impact, diabetes threatens the financial sustainability of healthcare systems by generating significant medical expenditures and long-term costs associated with permanent disability [2]. The significant increase in the incidence of DM and its complications persists despite considerable progress in therapies that not only reduce hyperglycaemia but also prevent chronic complications [3]. Current therapies still seem to fail, as patients do not properly implement them. Modifying lifestyle factors, such as adding more vegetables to the daily diet, increasing physical activity, or adopting Ayurvedic habits, could make them easier to adopt. Therefore, there is a need to develop new therapeutic methods in this area. Since oxidative imbalance is a key mechanism in the pathogenesis of hyperglycaemia and its chronic complications, this narrative review presents the latest research on the potential use of natural origin antioxidant compounds in the prevention and treatment of DM [4].

## 2. A Brief Overview of Current Concepts Regarding the Classification, Pathogenesis, and Treatment of Diabetes

### 2.1. Classification of Diabetes

DM is broadly classified into four primary categories based on its underlying pathophysiology and clinical characteristics. The two main types of diabetes—type 1 (T1DM) and type 2 (T2DM)—represent the most significant clinical and social problem.

T1DM is caused by the autoimmune destruction of pancreatic beta cells, resulting in an absolute deficiency in insulin production. T2DM results from a progressive loss of adequate insulin secretion that typically occurs alongside underlying insulin resistance (IR). Gestational diabetes mellitus (GDM) is defined as glucose intolerance that is first diagnosed during the second or third trimester of pregnancy. Finally, the classification includes “other specific types” of diabetes, which encompass conditions driven by distinct causes, such as genetic defects in beta-cell function (e.g., monogenic diabetes), diseases affecting the exocrine pancreas, such as cystic fibrosis, and medication-induced hyperglycaemia [5].

### 2.2. Pathophysiology of Diabetes

Thus, diabetes is a group of metabolic diseases characterised by hyperglycaemia resulting from a defect in insulin secretion and/or action. An example of diabetes in which insulin deficiency is the main mechanism leading to hyperglycaemia is T1DM. The pathogenesis of T1DM is driven by a chronic autoimmune assault that selectively destroys the insulin-producing β-cells in the pancreatic islets. This targeted destruction is initiated by a complex interplay between genetic susceptibility, environmental triggers, and defective immune tolerance [6]. Polymorphic alleles in the human leukocyte antigen (HLA) class II complex account for up to 50% of susceptibility to T1DM. Specific combinations of HLA alleles predispose to defects in central and peripheral immune tolerance, allowing autoreactive T-cells to escape normal regulatory checkpoints, e.g., programmed cell death protein 1 (PD-1) and cytotoxic T-lymphocyte-associated protein 4 signalling. These cells mistakenly identify native β-cell antigens as foreign, priming the immune system for a targeted attack against the pancreatic islets [7]. Loss of these inhibitory signals can facilitate autoreactive T-cell activity and β-cell destruction. This phenomenon is clinically demonstrated by immune-checkpoint-inhibitor-associated diabetes, an uncommon but serious endocrine immune-related adverse event occurring during cancer therapy with PD-1 or CTLA-4 blockade [8]. Exposure to environmental factors, including the gut microbiome, diet, and viral infections, is essential for triggering the autoimmune cascade. These environmental stressors can induce epigenetic modifications, such as altered DNA methylation patterns, which block cellular amplifiers in pro-inflammatory states and transform genetic risk into active disease [9].

On the other hand, T2DM, is characterised by a combination of peripheral IR and progressive pancreatic β-cell dysfunction. IR occurs when tissues—mainly skeletal muscle, liver and adipose tissue—show a reduced ability to respond to physiological levels of insulin circulating in the blood. In a healthy state, insulin promotes glucose uptake and inhibits hepatic glucose production, but in T2DM, these processes are disrupted. Initially, pancreatic β cells compensate for this IR by secreting larger amounts of insulin (hyperinsulinemia). However, over time, the constant demand leads to β-cell exhaustion, apoptosis, and a decline in insulin-secretory capacity, ultimately resulting in overt hyperglycaemia [10]. The main cause of IR is excess and dysfunction of adipose tissue, which occurs in the course of overweight and obesity. Adipocytes overloaded with energy stores lose their ability to accumulate lipids and shift the cytokine and adipokine profile toward a pro-inflammatory state. In addition, some of them die, which attracts immune cells and gives rise to metabolic inflammation. This inflammation is responsible for the development of IR in organs that are key to insulin function, such as the liver and muscles, as well as for pancreatic islet dysfunction leading to impaired insulin secretion [11]. Although the onset of T2DM is heavily influenced by environmental factors, there is also a strong genetic component, as individuals with a family history of the disease are at significantly higher risk [12]. Furthermore, emerging evidence suggests that disruptions in the gut microbiome contribute to the systemic hormonal and metabolic imbalances seen in these patients [13]. In consequence, altered short-chain fatty-acid production, impaired bile-acid transformation, increased intestinal permeability, and translocation of bacterial products such as lipopolysaccharide may contribute to metabolic endotoxaemia and chronic low-grade inflammation. These processes can disrupt insulin signalling in adipose tissue, liver, and skeletal muscle, while inflammatory stress within pancreatic islets may accelerate β-cell dysfunction [14].

### 2.3. Acute and Chronic Complications of Diabetes

Regardless of its pathogenesis, chronic hyperglycaemia is associated with damage, dysfunction, and failure of various organs, especially the eyes, kidneys, nerves, heart, and blood vessels. Diabetic complications are classically divided into acute metabolic disorders and chronic conditions affecting individual organs. Acute complications include diabetic ketoacidosis (DKA), hyperosmolar hyperglycaemic state (HHS) and severe hypoglycaemia, which, if left untreated, can lead to mental disorders, convulsions and coma. Chronic complications are broadly divided into microvascular diseases (diabetic retinopathy, nephropathy and neuropathy) and macrovascular diseases (coronary artery disease, cerebrovascular disease and peripheral artery disease) [15].

#### 2.3.1. Acute Complications of Diabetes

Acute metabolic complications occur when there is a critical imbalance between circulating insulin and counter-regulatory hormones. In DKA, absolute or relative insulin deficiency promotes lipolysis, hepatic ketogenesis, metabolic acidosis with a high anion gap, osmotic diuresis, and a severe reduction in body fluid volume. HHS is characterised by extreme hyperglycaemia, hyperosmolarity, profound dehydration, and minimal or no ketosis, usually affecting individuals with T2DM with preserved residual endogenous insulin secretion [16]. Severe iatrogenic or spontaneous hypoglycaemia, often occurring in patients treated with insulin or sulphonylureas, leads to neuroglycopenic symptoms and may progress to convulsions, coma, and permanent neurological damage [17].

Although acute metabolic complications of diabetes pose a direct threat to life, chronic complications are currently the main challenge for health care systems and the primary cause of deterioration in quality of life and reduced life expectancy among patients.

#### 2.3.2. Chronic Complications of Diabetes

Microvascular complications are primarily mediated by chronic hyperglycaemia–induced damage to small blood vessels. Diabetic retinopathy involves microaneurysms, capillary nonperfusion, and neovascularization, ultimately leading to macular oedema and vision loss [18]. Diabetic nephropathy is characterised by glomerular hyperfiltration, albuminuria, and a progressive decline in the estimated glomerular filtration rate (eGFR), which can culminate in end-stage kidney disease [19]. Diabetic neuropathy, including distal symmetric polyneuropathy and autonomic neuropathy, results in sensory loss, dysesthesia, and dysfunction of cardiovascular, gastrointestinal, and genitourinary systems [20].

Macrovascular complications reflect accelerated atherosclerotic changes in large and PKC medium-sized arteries caused by dyslipidaemia, endothelial dysfunction (ED), low-grade inflammation and prothrombotic conditions associated with diabetes [21]. Clinical signs include heart conditions (such as angina and heart attacks), brain blood flow issues (like mini-strokes and strokes), and narrowed leg arteries causing pain and reduced blood flow. In combination with peripheral neuropathy and impaired immunity, peripheral arterial disease contributes to the development of diabetic foot ulcers, infections and a high risk of non-traumatic lower limb amputation [22].

#### 2.3.3. Pathogenesis of Chronic Diabetic Complications

The primary trigger for the development of chronic complications of diabetes is hyperglycaemia, which activates several interconnected pathways in susceptible cells. The key pathogenetic pathways involved in the development of chronic complications of diabetes include the polyol and hexosamine pathways, protein kinase C (PKC) activation, and advanced glycation end-product (AGE) synthesis.

In chronic hyperglycaemia, excess glucose is reduced to sorbitol by aldose reductase, consuming nicotinamide adenine dinucleotide phosphate (NADPH) and generating osmotic stress. Since NADPH is needed for glutathione regeneration, its deficiency enhances OS (see Section 3) and impairs Na^+^/K^+^-ATPase activity in nerves, endothelium, and pericytes [23]. Hyperglycaemia also leads to excessive production of glucose-6-phosphate (G6P, a product of the first stage of glycolysis). G6P is then converted by glutamine-fructose-6-phosphate amidotransferase (GFAT) into glucosamine-6-phosphate, which causes increased O-GlcNAcylation of proteins and transcription factors, thereby activating pro-inflammatory pathways [24]. Moreover, diacylglycerol, formed as a result of conversion of glycerol-3-phosphate (a product of glycolysis), together with reactive oxygen species (ROS), activates PKC, thereby altering blood flow, increasing vascular permeability, thickening the basement membrane, promoting extracellular matrix deposition, inducing abnormal angiogenesis, enhancing leukocyte adhesion, and vascular cell apoptosis [25]. In turn, non-enzymatic glycation of proteins, lipids, and nucleic acids yields AGEs that cross-link extracellular matrix proteins (e.g., collagen) and bind to their receptor (RAGE) on endothelial and inflammatory cells, increasing activation of pro-inflammatory pathways, leading to excess synthesis of cytokines, adhesion molecules, and vascular endothelial growth factor (VEGF) [26].

**Figure 1:**
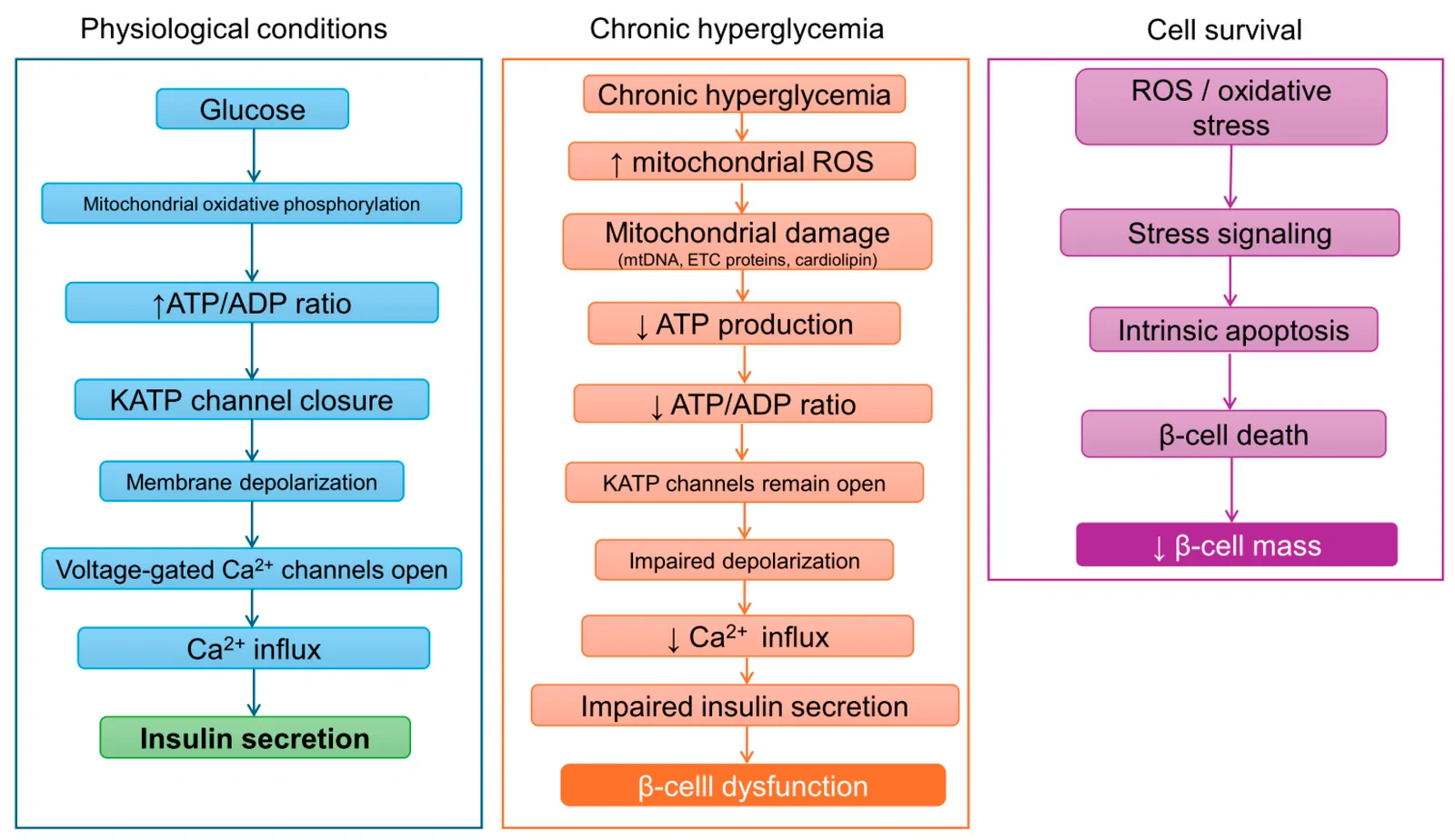
Glucose-stimulated insulin secretion and β-cell dysfunction under chronic hyperglycaemia and oxidative stress (OS). This diagram compares normal glucose-stimulated insulin secretion with β-cell dysfunction caused by chronic hyperglycaemia and OS. Excess mitochondrial ROS reduces ATP production, disrupts ion channel activity, impairs calcium influx, and leads to decreased insulin secretion and β-cell loss. ADP—adenosine diphosphate, ATP—adenosine triphosphate, Ca^2+^—calcium ion, ETC—electron transport chain, KATP channel—ATP-sensitive potassium channel, mtDNA—mitochondrial DNA, ROS—reactive oxygen species.

**Figure 2:**
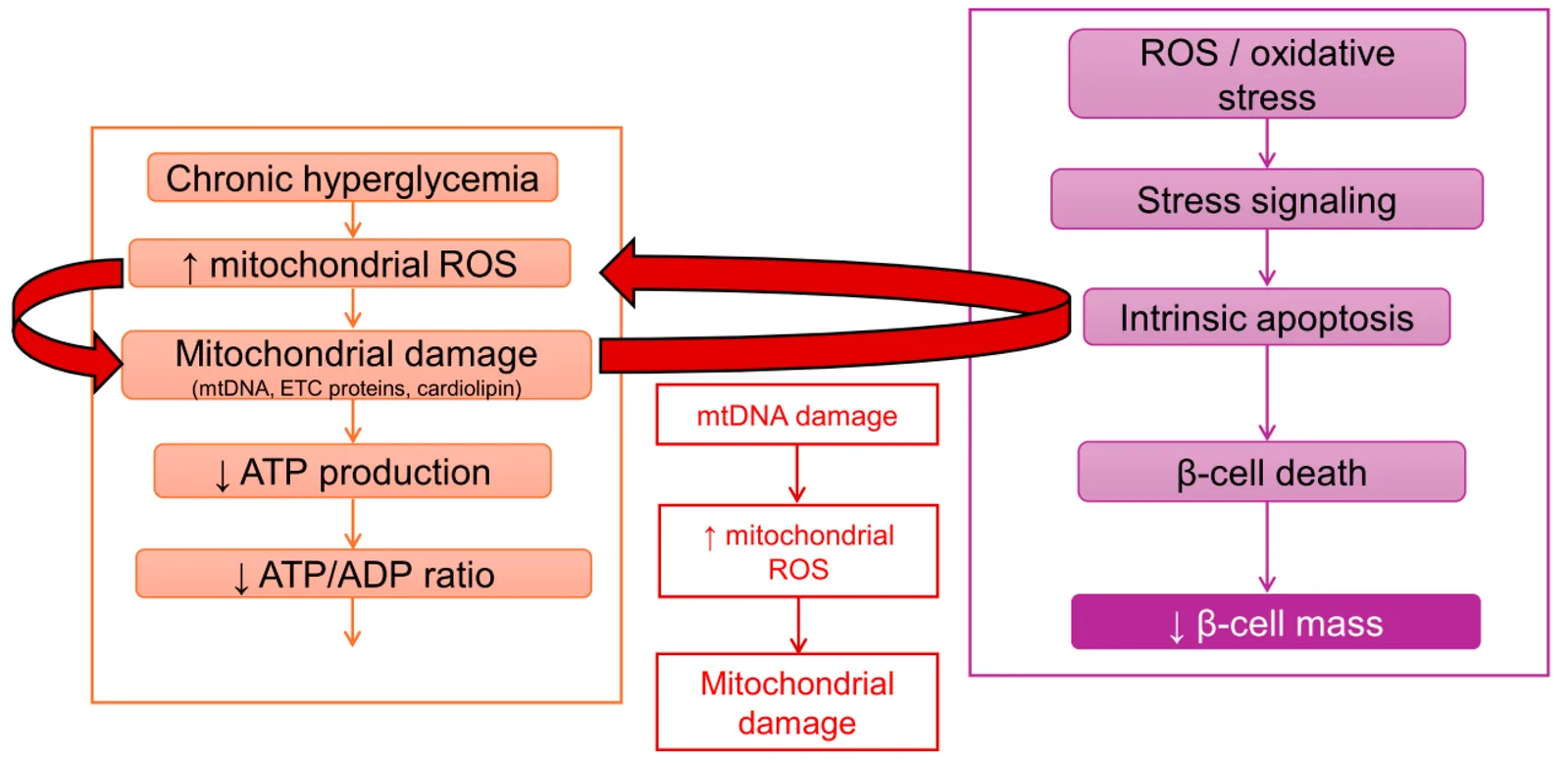
Vicious cycle of mitochondrial damage and ROS amplification. Mitochondrial damage increases ROS production, which further damages mitochondrial components (e.g., mtDNA and ETC proteins). This self-amplifying loop perpetuates oxidative stress, progressively worsening mitochondrial dysfunction and β-cell impairment. ADP—adenosine diphosphate, ATP—adenosine triphosphate, ETC—electron transport chain, mtDNA—mitochondrial DNA, ROS—reactive oxygen species.

In β cells, mitochondrial OS impairs both stimulus-secretion coupling and cell survival. Physiologically, glucose-stimulated insulin secretion depends on ATP production in mitochondria, which closes ATP-sensitive potassium channels, depolarises the cell membrane and opens voltage-dependent calcium channels (Figure 1). In the state of chronic hyperglycaemia, ROS-induced damage to mitochondrial DNA (mtDNA), respiratory chain proteins, and cardiolipin disrupts ATP synthesis, impairs ATP/ADP growth, and weakens insulin exocytosis, contributing to progressive β-cell dysfunction. Concurrently, prolonged oxidative damage activates intrinsic apoptosis pathways and abnormal stress signals, thereby reducing β-cell mass. These processes are amplified by a vicious cycle in which mtDNA damage further increases ROS production and, over time, causes mitochondrial dysfunction to spread (Figure 2) [27].

In turn, in insulin-sensitive tissues such as skeletal muscle, adipose tissue, and the myocardium, mitochondrial OS promotes IR and organ-specific complications. ROS modifies key components of insulin signalling, alters mitochondrial dynamics and biogenesis, and shifts substrate utilisation toward lipid oxidation, all of which interfere with insulin-stimulated glucose uptake [28].

In the diabetic retina, kidney, and neurons, high glucose increases mtDNA damage and downregulates genes encoding respiratory chain components, leading to a self-perpetuating loop of ROS generation, impaired repair, and cell loss, which together contribute to the development of microvascular complications: retinopathy, nephropathy, and neuropathy [29].

Even when blood sugar is well-controlled, tissue damage continues in people with diabetes. This “hyperglycaemic memory” occurs because ongoing reactive oxygen species production, gene shifts, and inflammation cause lasting harm after glucose levels normalise [30].

Targeted removal of hydrogen peroxide from mitochondria in experimental models improves insulin action and glucose tolerance and protects against the development of diabetic complications, highlighting the causal role of mitochondrial oxidants in multi-organ dysfunction in diabetes [31]. These observations have made mitochondrial ROS and redox-sensitive signalling attractive therapeutic targets, and current research is focusing on mitochondria-targeted antioxidants and agents that restore mitochondrial biogenesis and quality control as potential adjunctive strategies in the treatment of diabetes [32].

## 3. Role of Oxidative Stress in the Pathophysiology of Diabetes and Its Complications

### 3.1. Nitric Oxide, Free Radicals and Oxidative Stress Cascade

Physiological free radical formation is essential for cellular homeostasis. However, various mechanisms can trigger radical overproduction, depleting antioxidant reserves. This imbalance drives the development of diseases like diabetes and accelerates associated vascular complications [33]. Maintaining physiological homeostasis requires strict regulation of ROS levels. Excessive ROS production is a critical risk factor in the pathogenesis of various lifestyle diseases, such as hypertension, atherosclerosis, and diabetes, which form the core scope of this review. The concept of an imbalance between oxidant synthesis and antioxidant defences was famously described as “oxidative stress” by Sies and colleagues [34].

To make the OS state clearer, it is helpful to realise that the bodies of mammals constantly generate free radicals. If antioxidants fail to neutralise them, the body develops oxidative stress. Because reactive oxygen species (ROS) contain unpaired electrons, they remain highly volatile and reactive [33]. Physiological free radical formation is essential for cellular homeostasis. However, various mechanisms can trigger radical overproduction, depleting antioxidant reserves. This imbalance drives the development of diseases like diabetes and accelerates associated vascular complications [33].

#### Mechanisms of Oxidative Stress in Diabetes Induction

**Role of oxidative stress in diabetes induction and development—** ***NO*** **a main player?**

Since Ignarro’s Nobel Prize, *NO* has continued to be one of the most extensively studied molecules in biology, as highlighted by Aleksandrowicz et al. [35]. Endogenous *NO* is synthesised locally via the L-arginine cascade by *NO* synthases (NOSs), releasing picomolar amounts that act in a paracrine manner [36]. Three genes encode these NOS isozymes: neuronal NOS (nNOS/NOS-1), inducible NOS (iNOS/NOS-2) and endothelial NOS (eNOS/NOS-3) [37]. eNOS-dependent *NO* plays a crucial role in vascular regulation; it diffuses from the endothelium into the vascular smooth muscle (VSM), activating guanylyl cyclase and triggering cGMP (cyclic guanosine monophosphate) mediated VSM relaxation and vasodilation [35]. *NO* release rate in the vascular endothelium, depends on the actual activity of eNOS regulated by bioavailability of several cofactors, e.g., tetrahydrobiopterine (BH_4_). It is noteworthy that differences between males and females were demonstrated in the expression of NOS protein and *NO* availability and NOS, shown both in Sprague Dawley and spontaneously hypertensive rats (SHR). According to Komers and Anderson NOS activity is higher at the beginning of diabetes, which was indicated in the animal DM models [38].

Notably, hyperglycaemia causes a deficit in BH_4_, an essential cofactor for *NO* synthesis, leading to its oxidation to 7,8-dihydrobiopterin (BH_2_). When BH_4_ bioavailability is reduced, the eNOS dimer uncouples into monomers, causing enzymatic dysfunction. This impaired monomeric eNOS produces superoxide alongside *NO*, thus becoming a major source of ROS.

This situation may seem like a protective mechanism due to increased *NO* bioavailability. However, it ultimately has a negative effect because of excessive *NO* synthesis, which can drive an unchecked rise in ROS levels. Under diabetic conditions, NOS becomes overactive and produces peroxynitrite. These free radicals cause oxidative modifications that lead to the deterioration of regional circulation [39,40]. Simultaneously, the *NO* bioavailability (is an essential factor in the control of vascular resistance) was reported to be diminished, both in diabetic patients and in animal experimental diabetic models, as compared with normoglycaemic individuals.

As indicated earlier (see Section 2.3.2. Chronic complications of diabetes) the DM chronic complications mostly originates from ED and vascular complications.

Thus, endothelial damage and dysfunction leading to an increase in vascular resistance, hypertension, and proteinuria are a result of the changes in *NO* synthesis and bioavailability—in the condition of hyperglycaemia. The mentioned processes participate in or are a consequence of OS, which *per se* is also in charge of these negative phenomena.

Recent studies have demonstrated that hyperglycaemia-induced OS stems from excessive ROS. This overproduction is primarily fuelled by several metabolic pathways: elevated protein kinase C (PKC) activity, polyol pathway flux, hexosamine pathway activation, spontaneous glucose autoxidation, and the accumulation of advanced glycation end-products (AGEs) [41,42,43] and others listed in Table 1. OS arises when the production of reactive oxygen and nitrogen species (RONS) overwhelms the antioxidant system’s capacity for neutralisation. The primary line of defence consists of antioxidant enzymes, including superoxide dismutases (SOD), catalases (CAT), and glutathione peroxidases (GPx) and others (see Table 2), which actively inhibit the formation of reactive species within tissues [44].

OS—stemming from mitochondrial impairment and NADPH oxidase activation—exacerbates plaque vulnerability via lipoprotein oxidation and the attenuation of vasoprotective signalling. Recent literature extensively outlines the interplay between lipid metabolism, inflammation, and oxidative pathways in atherogenesis and plaque destabilisation [45,46].

### 3.2. Oxidative Stress and Insulin Resistance

Hyperglycaemia induces elevated oxidative stress, which is characterised by accelerated protein glycosylation, increased lipid peroxidation, and a concurrent depletion of antioxidant defences [47]. Here we put more stress on the IR phenomena, as it was outlined earlier, OS disrupts insulin signalling pathways, leading to IR and contributing to diabetes-related complications. Additionally, ROS can degrade pancreatic islet cells, resulting in diminished insulin secretion [48]. Elevated glucose levels stimulate multiple biochemical pathways, thereby increasing ROS production in diabetic states. Consequently, OS emerges as a disrupted cellular redox state characterised by ROS overaccumulation and impaired tissue antioxidant capacity. As stated by Cielecka et al. IR develops closely with OS and ED [49]. Together, these factors drive blood vessel inflammation and plaque buildup. These molecular changes disrupt blood vessel stability. They also create a lasting inflammatory state that increases heart and metabolic risks.

As previously discussed, IR is a persistent metabolic dysfunction defined by reduced tissue sensitivity to insulin. This triggers a compensatory surge in circulating insulin relative to existing glucose levels [50,51,52]. Clinically, IR is more than a metabolic marker; it represents a comprehensive risk profile encompassing multiple organ systems, including the liver, vasculature, kidneys, eyes, and neural pathways.

Antioxidant systems operate through distinct hierarchical mechanisms to mitigate cellular oxidative stress. The primary line of defence—comprising SOD, CAT and GPx—functions enzymatically to inhibit or suppress the initial formation of RONS within biological tissues. Sequentially, the secondary line of defence relies on non-enzymatic scavengers, including vitamins C and E, coenzyme Q, and reduced glutathione (GSH). These molecules directly neutralise active free radicals via electron donation, thereby reducing them to structurally stable intermediates with significantly lower cytotoxic potential [53].

#### 3.2.1. Insulin Resistance—A Trigger or Effect of Oxidative Stress?

By activating downstream signalling pathways, insulin elevates the vascular production of NO, endothelin-1 (ET-1) and ROS in the cardiovascular system. Chronically high blood insulin may induce vascular dysfunction through the over-secretion of these mediators [43]. The basic question is, whether those vasoactive factors are released at similar level/concentration (only shifted up), or are some of them more pronounced?

The IR development is closely linked to OS and ED; both contribute to vascular inflammation and atherogenesis (broadly speaking—vascular complications). These cellular changes damage blood vessels and cause ongoing inflammation, raising heart and metabolic risks. Therefore, insulin resistance acts as a core connection between metabolic problems, vessel damage, and neuronal degeneration [54,55,56].

Insulin helps blood vessels relax and widen. It does this by activating a specific cell pathway (the PI3K/Akt signalling) causing eNOS activation that increases a helpful NO and by lowering a harmful ET-1 release (antiatherogenic effect). Lowering other proteins like haem oxygenase-1 (HO-1), VEGF, and vascular cell adhesion molecule-1 (VCAM-1) also protects blood vessels by reducing inflammation and cell damage. Insulin via the Grb/Shc/MAPK pathway promotes proatherogenic ET-1 and PAI-1 expression, alongside cell migration and proliferation [47]. In diabetic endothelial dysfunction, insulin signalling is disrupted by protein kinase C beta (PKC-β) and nuclear factor kappa B (NF-κB) activation [49]. This resistance triggers excessive NOX2-derived superoxide, severely damaging the aortic wall and accelerating atherosclerosis [42]. Moreover, TNF-α and NF-κB amplify oxidative stress and insulin resistance through the IKK-β pathway, causing coronary endothelial dysfunction in T2DM [43].

Metabolic dysfunction and impaired glucose clearance characterise IR, driven by reduced cellular responsiveness in hepatic, adipose, and skeletal tissues [49]. This impairment stems largely from diminished expression and failed translocation of GLUT4, a vital terminal step in glucose uptake that remains distinct from upstream insulin signalling efficacy.

As stated by Cielecka et al., IR contributes heavily to cardiometabolic pathology via endothelial dysfunction, oxidative stress, and low-grade inflammation [49]. This mechanism positions insulin resistance as a critical bridge uniting metabolic, cardiovascular, and cognitive impairments.

IR disrupts the normal cell path (PI3K/Akt/eNOS signalling) that physiologically activates NO and keeps blood vessels healthy. At the same time, it overactivates a backup route (MAPK/ERK pathway) that causes blood vessels to tighten, raises blood pressure, and causes inflammation [57,58].

This imbalance creates a long-term, low-level inflammation that worsens whole-body insulin resistance and harms blood vessel walls [59]. High blood sugar accelerates this damage through various chemical pathways. Excess glucose overworks cell mitochondria. This leads to a flood of superoxide molecules, the creation of advanced glycation end products, and the constant triggering of the AGE-RAGE signalling pathway [49].

eNOS activity and NO availability, driving ED marked by poor vasodilation, high vascular tone, and pro-thrombotic changes. Mechanistic studies connect these defects in insulin receptor signalling to decreased NO and higher levels of vasoconstrictive factors such as ET-1 [60,61].

IR in the brain sparks a harmful cycle. High glucose and fat (free fatty acids) levels increase ROS. These molecules overwhelm antioxidant defences and hurt mitochondria (reduced oxidative phosphorylation). The resulting stress activates inflammatory signals like NF-κB, which damages proteins, harms membranes, and threatens neuron function and survival.

OS and inflammation bridge metabolic dysfunction with cognitive decline and vascular damage, illustrating the systemic health risks of IR. Preclinical and clinical data indicate that obesity-driven oxidative stress contributes to dysfunctional glucose transport (affecting glucose transporter function), elevated ROS, and heightened neuroinflammation. Pharmacological targeting of these OS pathways mitigates inflammation and preserves neuronal integrity, highlighting a promising therapeutic approach against metabolic-associated cognitive decline [45,62]. Nevertheless, based on the existing literature, defining the precise functional role of insulin resistance remains challenging

#### 3.2.2. Oxidative Stress—Not Always a Bad Player

As elegantly summarised by Gonzalez et al., physiological concentrations of reactive oxygen species serve essential regulatory roles in processes ranging from differentiation to autophagy [63]. Oxidative stress arises if ROS generation surpasses antioxidant capacity, resulting in cellular destruction and potential organ failure. In diabetes, hyperglycaemia intensifies ROS accumulation in both mitochondria and the cytosol, accelerating pathological damage.

## 4. Current Methods of Diabetes Treatment

Management strategies in diabetes integrate lifestyle modification, pharmacologic therapy, and, in selected cases, metabolic surgery, all together aiming at optimal control of glycaemia, blood pressure, body mass, and lipids to prevent the development of chronic complications. In T1DM, intensive, functional insulin therapy remains the primary treatment, whereas in T2DM, there has been rapid development of new therapeutic options in recent years [64].

Pharmacologic treatment in T2DM should follow a stepwise, patient-centred approach. Metformin is typically the first-line agent due to its efficacy, safety profile, and cardiovascular benefits. When monotherapy fails to achieve glycaemic targets, combination therapy is introduced, chosen based on comorbidities, risk of hypoglycaemia, body weight, and cost considerations. Agents with proven cardiovascular and renal benefits—such as sodium–glucose cotransporter 2 (SGLT2) inhibitors and glucagon-like peptide-1 receptor agonists (GLP-1 RAs)—are preferred for patients with established cardiovascular disease, heart failure, or chronic kidney disease. Additional options include dipeptidyl peptidase 4 (DPP-4) inhibitors, thiazolidinediones, and insulin therapy, when required, to address severe β-cell failure. New therapeutic approaches focus on novel pathways, including dual incretin receptor agonists and agents that modulate insulin sensitivity via the gut-liver axis or energy metabolism [65].

Interestingly, some antidiabetic drugs have been shown to exert antioxidant properties beyond their glucose-lowering effects, which may contribute to their cardiovascular and renal protection in T2DM. Given the role of chronic hyperglycaemia in generating OS, the ability of antidiabetic agents to modulate redox balance adds an important pleiotropic dimension to their therapeutic profile [32].

Metformin, which is the first-line, gold standard drug for type 2 diabetes, was found to reduce intracellular ROS generation and improve markers of OS, such as advanced oxidation protein products (AOPP) and AGEs, while enhancing plasma antioxidant capacity. Its antioxidant effects are partly mediated *via* activation of AMP-activated protein kinase (AMPK), modulation of the Nrf2–FOXO3 axis (nuclear factor erythroid-derived 2-related factor 2–Forkhead box O3 gene), and inhibition of NADPH oxidase-dependent ROS production [66]. In turn, pioglitazone, acting via peroxisome proliferator-activated receptor gamma (PPAR-γ) -related pathways, also exhibits antioxidant activity, reducing AGEs and AOPP while increasing enzymatic antioxidants such as SOD and paraoxonase [67].

Sodium-glucose cotransporter 2 (SGLT2) inhibitors suppress mitochondrial ROS, downregulate pro-oxidant enzymes, upregulate antioxidant systems (e.g., SOD, glutathione peroxidase), and attenuate AGE-RAGE axis activation and inflammation. Such effects are thought to underlie part of their cardiorenal benefits, independent of glucose control [68].

GLP-1 RAs and DPP4/CD26 (Dipeptidyl Peptidase-4) inhibitors can enhance endogenous antioxidant defences by upregulating Nrf2-driven pathways and enzymes, including CAT, SOD, and GPx, while decreasing markers such as malondialdehyde and oxidised LDL. Clinical studies show that administration of these drugs to patients with T1DM and T2DM leads to reductions in OS markers and improvements in endothelial function, suggesting that they have a protective effect against oxidative damage in vascular and metabolic tissues [69].

Given the role of OS in the pathogenesis of diabetes and its complications, the search for new therapeutic strategies with antioxidant effects is highly justified.

### 4.1. New Approaches to DM Treatment

According to the WHO Global Traditional Medicine Strategy for 2025–2034 [70], evidence-based complementary medicine has the potential to support conventional healthcare and more comprehensively address people’s health and well-being needs. The strategy envisions universal access to safe, effective, and people-centred traditional, complementary, and integrative medicine (TCIM).

Among traditional medical systems, Ayurvedic medicine warrants particular attention. Ayurveda is one of the oldest medical systems in the world, having been developed over 5000 years. Nowadays, Ayurveda has been increasingly accepted and recognized as an alternative medicine in major countries across the OECD (Organisation for Economic Co-operation and Development) [71]. Many plants used in Ayurvedic medicine exhibit a broad spectrum of biological activities, including anti-inflammatory, antioxidant, hypoglycaemic, and immunomodulatory properties.

In the context of chronic diseases, such as diabetes, the search for new natural bioactive compounds is of particular importance. Many modern drugs are of plant origin or have been developed based on natural compounds, as exemplified by metformin; therefore, research on Ayurvedic botanical materials may contribute to the discovery of new therapeutic candidates.

#### 4.1.1. Characteristics of Three Plant Species with Antidiabetic and Antioxidant Properties: Bitter Melon (*Momordica charantia*), Gurmar (*Gymnema sylvestre*) and Jambolan (*Syzygium cumini*)

Among the Indian-origin botanicals we selected, these three have traditionally been used in the treatment of diabetes and are now supported by a growing body of scientific evidence and mechanistic studies. Their therapeutic potential is attributed to a wide spectrum of bioactive compounds, which exhibit a range of biological activities, including antioxidant, anti-inflammatory, and antidiabetic effects. Below, we present studies investigating extracts of these plants, as well as studies focusing on specific bioactive compounds isolated from them. A summary table is also provided, compiling the major groups of biologically active compounds identified in the selected plants and highlighting those that, based on the available evidence, have demonstrated potential antidiabetic activity (Table 3).

##### Bitter Melon—*Momordica charantia* L.

Commonly known as bitter melon, bitter gourd or sopropo, *Momordica charantia* L. (*MC*) belongs to the *Cucurbitaceae* family and is widely distributed in tropical and subtropical regions. Its fruits are commonly consumed as a vegetable in Southeast Asia. Additionally, *MC* tea, known as gohyah or herbal tea, is prepared from dried fruit slices and has traditionally been used for a variety of medical purposes, including antidiabetic, anthelmintic, antimalarial, and laxative applications. It has also been employed in the management of conditions such as dysmenorrhea, eczema, gout, jaundice, psoriasis, and rheumatism [72].

Analytical studies have revealed that water constitutes the major component of the *MC* fruit (93%), while polysaccharides and proteins account for approximately 30% and 18% of the dry weight, respectively. Polysaccharides are among the most important bioactive compounds isolated from *MC*. Their structural characteristics have been extensively investigated, showing that properties such as uronic acid content, monosaccharide composition, and molecular weight influences their biological activity. The polysaccharides are mainly composed of monosaccharides including arabinose (Ara), rhamnose (Rha), galactose (Gal), glucose (Glu), mannose (Man), xylose (Xyl), galacturonic acid (GalA), and glucuronic acid [73].

Among the proteins isolated from *MC*, the most extensively studied belong to the family of ribosome-inactivating proteins (RIPs), including lectins, momordins, momorcharins, and the *Momordica* anti-HIV protein (MAP30) as well as polipeptide-P [72]. Moreover, the globular seed fraction of *MC* mainly consists of globulins—high-molecular-weight proteins once considered inactive but now known to have various biological functions. These proteins belong to the cupin superfamily and are classified into 11–12S legumins and 7–8S vicilins. Vicilins are trimeric proteins with a high structural diversity. Their expression increases under dehydration, suggesting a role in protecting against desiccation and OS [74].

In addition to primary constituents, *MC* contains a diverse range of secondary metabolites, including triterpenoids (particularly cucurbitane-type triterpenoids and their glycosides), saponins (e.g., goyasaponins I–III), phenolic acids, flavonoids (e.g., quinic acid and catechin), essential oils, fatty acids, sterols, vitamins (C, A, E, B1, B2, B3, and B9/folate). Most of them have antioxidant activities by scavenging hydroxyl, superoxide and hydrogen peroxide radicals and having reduced power ability. It is noteworthy that *MC* also includes minerals (potassium, calcium, zinc, magnesium, phosphorus, and iron) [72].

##### Antidiabetic Properties of *M. charantia*

A substantial body of evidence indicates that bioactive compounds derived from *MC* possess hypoglycemic properties (e.g., reduced FPG (fasting plasma glucose), HBA1c (Hemoglobin A1c) and PPG (postprandial glucose) levels) demonstrated in animal models of diabetes [75,76] as well as in prediabetic and T2DM human populations.

The results of human studies indicate that *MC* is generally well tolerated. However, the reliability of the comparison of results and the ability to draw conclusions from them is limited. The studies were conducted on patients with diabetes as well as on prediabetics.

A meta-analysis comprising ten studies (combined *n* = 1045) indicated that *MC*, when taken orally at a dose of 2 to 6 g/day for at least four weeks, can reduce elevated blood glucose levels in patients with T2DM [77]. However, the authors highlighted the presence of low-quality evidence regarding the botanical material used, the composition of the preparations, the wide dosage range, and the small sample size in all trials. Furthermore, the majority of the included studies did not provide an adequate report of the safety data.

A recent meta-analysis of nine randomised, parallel-group, placebo-controlled trials has provided new insights into the existing evidence regarding the potential efficacy of *MC* in the treatment of metabolic syndrome. The analysis revealed that *MC* mono herbal preparations do not exert a significant positive influence on blood glucose levels or other cardiovascular risk factors associated with metabolic syndrome. However, *MC* was found to be statistically significantly more effective than a placebo in terms of reducing HbA1c and (marginally) fasting glucose levels when post-intervention data were analysed [78].

In order to minimise the influence of taking different types of medications and different disease histories in diabetic patients, a 12-week intervention study was conducted. The study assessed the effects of low-dose (300 mg/day) and high-dose (600 mg/day) of *MC* extract on HbA1c, related glycaemic measures, blood lipids and body composition in subjects with prediabetes. The *MC* groups showed relatively small increases in fasting blood glucose levels at 6 and 12 weeks, compared to the control group.

Additionally, as the authors concluded, the statistical power was low overall, and the meta-analysed studies were too short to reliably detect long-term MetS effects. It should be noted that MetS consists of a group of risk factors for cardiovascular disease and diabetes. However, treating individual components may be easier effective than targeting the syndrome as a whole. This highlights the need for additional research on this plant using carefully planned clinical trials of longer duration. However, a lack of efficacy in short-term studies does not necessarily mean a lack of efficacy with long-term use of *MC*. The limited sample size should also be considered when interpreting this finding. To confirm the clinical efficacy of *MC*, future randomized controlled trials require larger sample sizes, longer follow-ups, and dose-response evaluations in humans.

Concerns regarding discrepancies in plant genotypes, cultivation practices, environmental conditions, plant material, preparation methodologies, and dosage consistently emerge during the comparison of studies, thereby emphasising the importance of accounting for these variables in future research, including through the standardisation of used extracts.

The importance of plant material type and cultivation location was demonstrated in a recent study by Koopmans et al. on piglets [79]. In the adult STZ induced diabetic obese pig model for human obese metabolic syndrome and mild T2DM, the fruits of *MC* improve glycaemic control, whereas stems-leaves increase plasma insulin concentrations. Furthermore, the observed effect was cultivar-specific. This study may explain the varying results of human trials reported in the literature.

Although the precise mechanisms underlying the hypoglycaemic properties of *MC* observed in pre- and clinical studies remain to be elucidated, current studies suggest several potential pathways.

**Figure 3:**
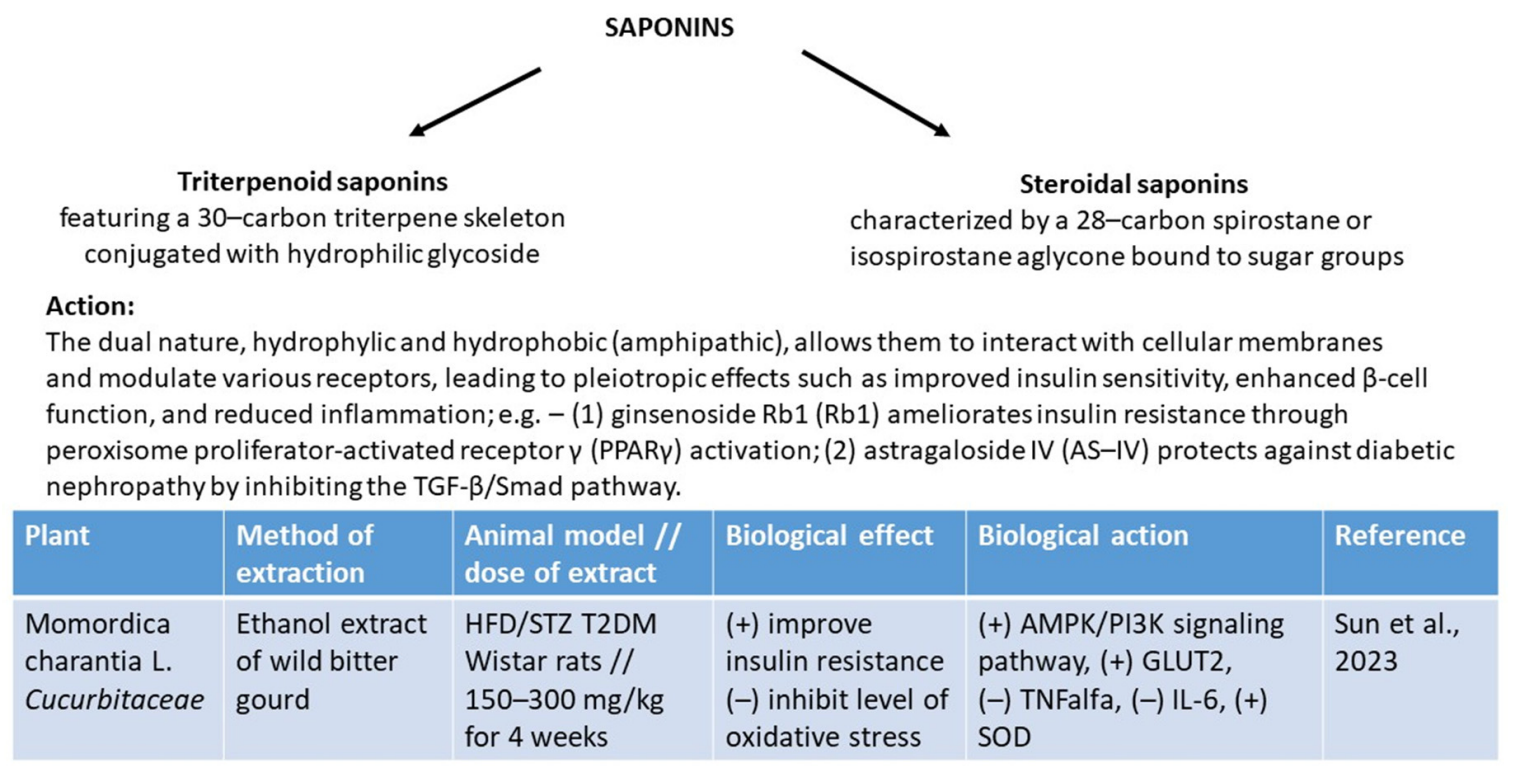
Scheme showing the chemical division of saponins according to their chemical structure (based on [84]).

It is evident that the antioxidant properties of *MC* are amongst the most well-documented and prominent of these. Antioxidant properties have been demonstrated in both in vitro and in vivo studies for polysaccharides [75,76,80,81], proteins [74], triterpenoid compounds [82] and saponins [83] extracted from the plant (Figure 3).

##### Antioxidant Mechanisms of *M. charantia*

It was shown that isolated polysaccharides from *MC* (MCPs) affect the Notch signalling pathway, which in turn influences the regeneration of pancreatic islets, leading to increased insulin synthesis and contributing to a reduction in blood glucose levels. These MCPs mainly consist of galactose, galacturonic acid, xylose, rhamnose, mannose [85]. Notch signalling is an intercellular communication pathway which regulates cell fate in cell type- and context-dependent mechanisms [86]. It has been demonstrated that Notch signalling plays a role in the regeneration of pancreatic islets in rats with diabetes induced by intraperitoneal administration of STZ, which were treated with *MC* extract. An increase in mRNA levels was observed for key regulators, including pancreatic and duodenal homeobox 1 (Pdx1), a crucial factor in pancreatic development and β-cell function; insulin 1 (Ins1), a gene encoding insulin, indicating improved β-cell function; Jagged1, a ligand of the Notch signalling pathway and Hes1, a Notch-dependent transcription factor. At the same time, a decrease was noted in the expression of genes associated with inhibition of islet regeneration, namely Notch1 and its activating ligand Delta-like ligand 4 (Dll4). Additionally, a reduced Bax/Bcl2 (Bcl-2-associated X protein/B-cell lymphoma 2) ratio was observed, indicating decreased apoptosis in pancreatic cells [87]. It is worth noting that the influence on Notch signalling has been demonstrated for many groups of biologically active plant compounds, such as flavonoids (e.g., quercetin), polyphenols (e.g., gallic acid), and terpenoids (e.g., curcumin, lupeol). This was studied in relation to cancer therapy, but significant anti-inflammatory effects have also been indicated in studies using models of inflammatory diseases [88]. Notably, *MC* leaves ethanolic extracts are a rich source of flavonoids, including quercetin and catechin or polyphenols (gallic acid) [89].

As mentioned earlier (see Section 3.1), ROS originate as transient, highly reactive intermediates from exogenous exposure or endogenous metabolic processes. The steady-state equilibrium of the cellular redox environment is dictated by cell type, as specialised populations differ significantly in their antioxidant scavenging kinetics and differential modulation of ROS flux. The steady-state equilibrium of the cellular redox environment is dictated by cell type, as specialised populations differ significantly in their antioxidant scavenging kinetics and differential modulation of ROS flux. When the production of ROS is imbalanced, it induces OS, which may cause chronic inflammation by damaging biomolecules [90]. Nrf2 (nuclear factor erythroid-derived 2-related factor 2) functions as a pivotal cellular defence mechanism against oxidative stress-induced damage. Following activation, this basic leucine zipper transcription factor binds to target promoter antioxidant responsive elements (ARE), upregulating cytoprotective genes necessary to restore and maintain cellular homeostasis [91]. Cellular homeostasis is tightly regulated by ROS within the stem cell niche. Fluctuations in ROS concentrations modulate the Nrf2-Notch1 signalling pathway to dictate stem cell fate. Initially, an elevation in ROS levels stimulates quiescent stem cells to undergo self-renewal, facilitating tissue repair. Subsequently, the accumulation of endogenous antioxidants diminishes ROS concentrations. This negative feedback loop suppresses further proliferation, thereby preventing hyperplastic growth that could compromise the structural integrity of the regenerating tissue [92]. Thus, in stem cells, the ROS-Nrf2-Notch pathway was suggested to be an evolutionarily conserved mechanism that allows stem cells to adapt to fluctuations in the level of ROS in their environment [93].

Nrf2 was shown to be in the middle of the complex regulatory network of antioxidant polysaccharides. Under OS, the key transcription factor Nrf2 induces the expression of various antioxidant enzymes. It operates within signalling pathways such as Keap1/Nrf2/ARE, PI3K/Akt/GSK3β, JNK/Nrf2, and NF-κB. Upon nuclear translocation, Nrf2 activates downstream antioxidant gene expression, thereby mitigating oxidative damage. By modulating these Nrf2-mediated pathways, polysaccharides maintain homeostatic Nrf2 expression within the body. It was shown that administration of highly pure MCPs from dried *MC* slices in the aging rat model showed that aging-induced neuronal injury is mitigated by MCP administration, which drives the nuclear import of Nrf2 and β-catenin [94]. This suggests the Nrf2/β-Catenin signalling pathway. Current evidence indicates that the Wnt/β-catenin signalling pathway might be a key regulator of glucose homeostasis. Disruption to this pathway may increase diabetes susceptibility, influencing not only β-cell proliferation and total mass, but also regulating tissues that control appetite, energy expenditure, and growth [95]. It was found that processing conditions including extraction method, drying methods and temperature can affect the content and antioxidant capacity of active compounds including structures and biological activities of polysaccharides [73,96]. This brings up the question of how to standardise and compare the effectiveness of various extracts.

In the context of the recent results the antioxidants contained in the plant (e.g., ferulic and cinnamic acids or vanillin and vanillic acid) may also act by inhibiting inflammation (an important element in the pathogenesis of diabetes, vascular dysfunction and in the end vascular complications, broadly speaking) where increased levels of free radicals play a key role [89,97]. Scavenging free radicals suppresses inflammation at its source.

Considerable evidence links low-grade inflammation to IR development. Inflammatory cytokines, namely interleukin-6 (IL-6) and TNF-α, impair insulin signalling and glucose uptake in muscle and adipose tissues, alongside promoting hepatic gluconeogenesis and lipolysis. Mechanistically, chronic inflammation drives ROS accumulation, which further compromises insulin signalling and cellular degradation.

MCP has been shown to protect the rat myocardium from isoproterenol-induced apoptosis by lowering intracellular ROS, reinstating endogenous antioxidant enzymes, and downregulating pro-apoptotic proteins; these actions subsequently inhibit the inflammatory cytokines IL-6 and TNF-α [81]. Moreover, cytokines cause OS via activation of immune cells, namely macrophages, which produce ROS to clear pathogens. During T2D and chronic inflammation, macrophages change from a healing (M2—anti-inflammatory) state to an attacking (M1—pro-inflammatory) profile. This shift helps start and worsen inflammation in the pancreas [98]. *MC* was found to reduce the activity of inflammatory genes like IL-1β, IL-6, IL-10, iNOS, COX-2 and TNF-α in macrophages treated with lipopolysaccharide (LPS) [99]. It was shown that this activity of *MC* may be linked to charantin (β-sitosteryl glucoside), an important bioactive compound found in the plant fruit, known primarily for its anti-diabetic property [99,100].

Additionally, *MC* fruit extracts contain several other antioxidant compounds, including ascorbic acid, brevifolincarboxylic acid, cucurbitacin E, goyaglycoside G, kuguacin H, 3-malonylmomordicin I, margarolic acid and quercetin 3-O-glycoside [101]. In addition to the previously mentioned compounds, various antioxidant substances from different sources have been identified in the ethanolic extracts of *MC* leaves, such as ferulic and cinnamic acid (hydroxycinnamic acid group), gallic and vanillic acid (hydroxybenzoic acid group) and vanillin (phenolic aldehyde) [89].

#### 4.1.2. Gurmar—*Gymnema sylvestre*

Known as “Gurmar” (means “sugar destroying”) and also Gymnema or periploca of the woods, Gymnema sylvestre (*GS*), belongs to the family of *Apocynaceae* and it is a wild herb found in India, Africa, Australia and China. *GS* was considered as one of the major botanicals to treat diabetes and various inflammatory diseases in the folk and Ayurvedic system of medicine [102]. Different formulations of this plant are widely used in a number of preparations such as tea bags, health tablets, and food supplements.

##### Antidiabetic Properties of *G. sylvestre*

The most widely recognised effect of *GS* is its antidiabetic activity. A number of animal studies indicated a reduction in glucose levels following administration of *GS*. The main constituents responsible for antidiabetic activity of *GS* are gymnemic acids, which are triterpenoid saponins (pentacyclic molecules with a broad spectrum of biological and pharmacological activities) [103]. Their ability to reduce elevated levels of blood glucose and increase plasma insulin were reported in STZ-diabetic mice [104]. In addition, *GS* contains several other constituents such as alkaloids, anthraquinones, chlorophylls, flavones, quercitol with potential antidiabetic properties, which were also shown to mitigate the inflammatory markers [105].

When the whole plant extracts were studied the most pronounced hypoglycaemic effect was observed in response to the ethanol extract [106,107,108,109,110]. *GS* exhibited pleiotropic effects, including modulation of multiple metabolic parameters and improvement of liver and kidney function. In dexamethasone-induced IR rats, treatment with aqueous extract of *GM* significantly altered the elevated glucose, lipid, insulin levels and increased antioxidant capacity, resulting in improved histology of the liver [111]. In another study where alloxan-induced diabetic rats were used, this plant extract reduced fasting blood glucose level, total cholesterol, serum triglycerides and increase HDL-cholesterol level and is also described to alter the elevated level of urea, uric acid and creatinine levels in diabetic rats to nearly normal levels [112,113]. However, Galletto et al. also informed about an absence of antidiabetic activity of *GS* in an alloxan treated animal model [114]. Although the diabetes models and the experimental protocol were similar, the authors tested a single dose [112,114] or three different doses [113] of dried powdered GS leaves [114] or an aqueous leaf extract of the plant [112,113]. Possibly, the main reason for the different results was that aqueous extracts exhibited higher activity.

Despite the encouraging outcomes observed in studies on animal models of diabetes, there is a limited number of clinical studies investigating the effects of *GS* in humans with diabetes or metabolic disorders. In patients with obesity, *GS* effect on fasting glucose and adipokines related to IR was better than treatment with berberine, with minimal side effects reported [115]. *GS* administration in patients with IGT (i.e., impaired glucose tolerance) decreased 2-h OGT (oral glucose tolerance) test, glycated haemoglobin (HbA1c), and increased insulin sensitivity. There were also improvements in anthropometric measures and the lipid profile [116]. In patients with insulin-dependent diabetes under insulin therapy *GS* supplementation appears to decrease serum lipids to almost normal levels and enhance endogenous insulin, possibly by regeneration/revitalisation of the residual beta cells in insulin-dependent diabetes mellitus [117].

In vitro studies using isolated mouse and human islets of Langerhans have also demonstrated the direct stimulatory effects of an aqueous alcohol extract of fresh *GS* (OSA^®^) on insulin secretion from beta cells [118], which is at least partly due to Ca^2+^ influx and protein kinase activation [119]. Moreover, OSA^®^ was also reported to significantly stimulate insulin production and maintain intraislet insulin stores in mouse islets by promoting PPI mRNA levels. Furthermore, it was found to protect β-cells from apoptosis by reducing cytokine-induced caspase-3/7 activity in MIN6 cells (mouse insulinoma 6 cells) and mouse islets, while increasing antiapoptotic effectors.

The ability to increase insulin production in pancreatic β-cells in vitro was demonstrated for the triterpene glycoside fraction that was isolated and purified from the ethanolic extract of *Gymnema sylvestre* [120]. When studying the effects of different plant substances, it is also important to consider whether the observed effects, although statistically significant, are also clinically relevant. In the above-mentioned study of Shenoy et al., the effect of the triterpene glucoside fraction from *GS* in stimulating insulin secretion was comparable to that of glibenclamide, a standard drug for this purpose [120].

##### Antioxidant Properties of *G. sylvestre*

Shenoy et al. noted that, when investigating the mechanisms underlying decreased insulin secretion and β-cell damage in diabetes, the vulnerability of pancreatic cells to ROS was identified as a key pathological factor [120]. They therefore studied H_2_O_2_ as a well-established model of biological ROS, demonstrating significant reductions in ROS formation at all concentrations of the triterpene glycoside fraction isolated from *GM* compared with the control. Vasi and Austin (2009) showed that *GS* administration not only exerted hypoglycaemic activity but also significantly reduced plasma lipid peroxide levels and increased SOD activity and serum albumin content in diabetic rats [121]. The increase in albumin levels may also contribute to protection against OS through albumin’s antioxidant properties and its ability to bind reactive molecules.

The antioxidant effects of *GS* have also been demonstrated in other experimental models [122,123,124].

Similarly to the previous plant, the content of active compounds depends both on its genotype and on the method used to prepare the extract. It should be emphasised that very recent studies indicate that methanol and water were more efficient extraction solvents for the whole plant of *GS* than ethanol, producing higher extraction yields. All solvent extracts contained important phytochemicals such as alkaloids, tannins, flavonoids, phenolics, and terpenoids, while glycosides were detected only in ethanol and water extracts, suggesting that solvent polarity affects their extraction. Saponins were highly positive in water but negative in ethanol whereas proteins were negative in methanol but positive in ethanol and water extracts. Ethanol proved to be the most effective solvent for extracting flavonoids and tannins. However, the methanol extract demonstrated the strongest overall antioxidant activity and the lowest IC_50_ value, indicating the highest antioxidant potential. Since the IC_50_ values of all extracts were relatively similar, the plant appears to possess moderate antioxidant activity regardless of the solvent used. The results also suggest that antioxidant effects are likely due to the combined action of phenolics, flavonoids, tannins, and terpenoids. Overall, the findings highlight how solvent polarity strongly influences phytochemical composition and biological activity of plant extracts [125].

#### 4.1.3. Jambolan—*Syzygium cumini* (L.) Skeels

Commonly known as jambolan or black plum, ***Syzygium cumini*** **(L.)** **Skeels**, (former *E. jambolana* Lam.) is also known as Java plum, Malabar plum, jamun, Indian blackberry, Portuguese plum, Malabar plum, purple plum, Jamaica and damson plum. This plant is a member of the *Myrtaceae* family and is commonly found in the Indian subcontinent, Southeast Asia, Queensland, Pakistan, Bangladesh and China. *S. cumini* fruits are eaten fresh, or as chutney, and jam. *S. cumini* (*SC*) juice is used for preparing summer drinks such as syrup or sherbet. Various extracts of jambolan isolated from different parts of plants, including the roots, stems, leaves, flowers, and fruits possess a range of pharmacological actions such as antibacterial, antifungal, antiviral, antigenotoxic, anti-inflammatory, antiulcerogenic, cardioprotective, antiallergic, anticancer, chemopreventive, radioprotective, free radical scavenging, antioxidant, hepatoprotective, antidiarrhoeal [126]. Interestingly, due to their antioxidant and antimicrobial properties, they are used commercially as preservatives in many foods, particularly in meat processing, as an alternative to chemical preservatives. They have also been used in various industrial applications and are a common ingredient in perfumes and soaps, as well as being used as a cleansing agent in histological work [127]. *SC* seeds are used in Siddha, Ayurveda and Unani medical systems as a diabetes remedy.

Recent publications confirmed that different *SC* plant parts have different and characteristic compositions. According to the database collected by Qamar et al. (2022) seeds contain: fatty acids, phenolic acids, flavonoids and tannins; leaves are rich in: alkanes, terpenoids, flavonoids, phytosterols [126]. Stem bark contains: terpenoids, phenolic acids, flavonoids; flowers: flavonoids terpenoid (oleanolic acid) and phenolic acid (ellagic acids), whereas essential oils are a mixture of terpenoids. Antioxidant properties of *SC* have been confirmed in many studies for different parts of the plant. In particular, for fruits all studied cultivars showed high+ 90% DPPH (2,2-diphenyl-1-picrylhydrazyl) scavenging ability, which may be attributed to the collective participation of several antioxidant components such as ascorbic acid, total phenols, and total anthocyanins, all present in high contents [128,129,130,131]. Substantial differences in the number of anthocyanins were reported among the studies, and it was suggested that different geographical regions and climatic conditions could potentially explain these differences, as well as the extraction procedure and drying method of the pulp.

Several studies have investigated the antioxidant activity of leaves, seeds, and stems using different extraction solvents, including ethanol and water. Ethanolic extracts of leaves, seeds, and stems exhibited markedly higher free radical scavenging activity, removing 68%, 98%, and 33% of free radicals, respectively, compared with aqueous extracts, which showed activities of 5.09%, 1.25%, and 7.26%, respectively. Ascorbic acid, used as a reference standard, demonstrated 97% free radical scavenging activity [132].

In another study comparing seeds with fruit pulp, seed extracts exhibited approximately twofold higher oxygen radical absorbance capacity (ORAC) values. Seed extracts also showed stronger ABTS (i.e., radical cation ABTS+) radical scavenging activity than DPPH scavenging activity, achieving 50% radical inhibition at substantially lower concentrations. According to the authors, the higher ORAC values observed for SC extracts may be attributed to their elevated total polyphenolic content, consistent with the reported linear relationship between ORAC values and total phenolic content [133]. Furthermore, ellagic acid concentration in seed extracts was nearly 20-fold higher than in pulp extracts [133]. However, fruit skin was reported to contain the highest levels of bioactive compounds and antioxidant capacity, followed by pulp and seeds [134].

Additionally, seed extracts showed antioxidant properties in animals indicated by decrease in LPO (lipooxygenase) and increase in GSH levels in the gastric mucosa of rats with gastric ulcers [135] as well as significantly reduced plasma lipid peroxide levels in diabetic rats. Treatment with the extracts also increased the activity of the antioxidant enzyme SOD as well as serum albumin levels [121].

Antioxidant properties have also been reported for bark and leaf extracts of *Syzygium cumini*. In studies evaluating bark extracts, the efficiency of extracting antioxidative compounds decreased in the following order: ethanol, methanol, and acetone, indicating that ethanol was the most effective solvent for the recovery of antioxidant constituents [136].

Furthermore, aqueous leaf extracts of *SC* demonstrated strong protective effects against OS. The extracts were shown to inhibit the excessive generation of RONS, prevent macrophage viability loss, and reduce foam cell formation induced by LDL oxidation [137].

##### Dependency of *S. cumini* Antidiabetic Properties on the Part Tested: Seeds, Fruits or Leaves

The antidiabetic properties of various plant parts have been studied in animal models of experimentally induced diabetes. Unfortunately, the number of available clinical studies remains very limited.

Seeds. *SC* seeds have been known and used for centuries as a remedy for diabetes. The largest body of evidence indicates that, whether in powder or extract form (ethanol, methanol, ethyl acetate, and water) and administered by various routes, seeds affect glycaemic control [126]. In particular, the hypoglycaemic and hypolipidemic effects of the ethanolic extract of seeds were confirmed in alloxan-induced diabetic rabbits [138], rats [139,140] and mice [141], as well as in STZ-induced diabetic rats [142]. In rabbits, the oral administration of *SC* water and methanol extracts resulted in almost the same hypoglycaemic effect as that achieved with the standard drug tolbutamide, which shows the clinical value of *SC* [138].

In humans, Sahana et al. reported that *SC* seed in newly diagnosed T2DM patients decreased fasting blood sugar, HOMA-IR, and increased HDL value at the third month of the study [143]. However, these beneficial findings do not translate into a decrease in postprandial blood sugar or HbA1c at any point during the study. Authors concluded that fasting blood sugar results from increased hepatic glucose production, which is inhibited by relatively low insulin levels; higher levels are needed for maximal stimulation of peripheral glucose uptake, i.e., to decrease post-meal glucose. This shows that *SC* may be only a weak insulin secretagogue, if at all.

Fruits. The oral administration of the fruit-pulp extracts reduced fasting blood glucose and improved blood glucose in the glucose tolerance test in healthy and alloxan-induced mild and severely diabetic rabbits. For the fruit of *SC* water extract of fruit pulp was found to be more effective than the ethanolic extract and more effective than tolbutamide (250 mg/kg, body weight) [144]. Since water extract of fruit pulp showed activity even in severe diabetic rabbits in which most of the islet cells are damaged, it is likely that water extract of pulp is also having some direct effect on the tissue utilisation of glucose. However, Pepato et al. in a study investigating the effects of lyophilised fruit pulp extract administered for 41 days in STZ induced diabetic rats, observed no significant differences in body weight, food and water intake, urine volume, glycemia, urinary urea and glucose levels, hepatic glycogen content, or serum total cholesterol and triglyceride concentrations [145].

Leaves. Texeira et al. performed experiments with healthy rats, rats with STZ-induced diabetes, volunteers, and patients with diabetes and found no antihyperglycemic effect of *SC* crude extract and tea prepared from leaves [146].

##### Antioxidant Mechanisms of *S. cumini*

Plasma insulin levels increased in diabetic rats [147] and in both diabetic and severely diabetic rabbits treated with fruit pulp extract. In vitro studies with pancreatic islets showed increased insulin secretion after administration of fruit pulp extract [144,145]. In the pancreatic islets of diabetic rabbits, insulin release was nearly 2.5 times that in untreated diabetic rabbits [144]. Moreover, the inhibition of insulinase activity in the liver and kidney by the *SC* extract has been reported [147].

It has been well established that the plant’s active compounds have strong antioxidant effects, which underlie the aforementioned protective effect and the inhibition of inflammatory processes, which was confirmed both in vitro and in vivo studies [126]. The most thoroughly studied are seed extracts and, to a certain degree, fruit. They are rich in phenolic acids, flavonoids, tannins (including proanthocyanidins), and fatty acids, and have a proven inhibitory effect on alpha-glucosidase and alpha-amylase, increase glycogen synthesis, and possess a strong antioxidant effect.

Diabetes-related OS changes and the antioxidative effect of *SC* seed extract administration were reported by Vasi and Austin in STZ-induced diabetic rats [121]. They observed elevated plasma lipid peroxide levels in diabetic rats along with a significant decrease in the antioxidant enzyme, SOD activity as well as reduced levels of serum albumin and uric acid confirming that diabetes and its complications are associated with free radical mediated cellular injury. *SC* seed extract administration not only has hypoglycaemic activity but it also significantly reduces plasma lipid peroxide levels in diabetic rats. Moreover, following the treatment with *SC*, the activity of the antioxidant enzyme SOD and serum albumin content were also increased. However, the serum uric acid content was not found significantly altered. SOD and albumin form the primary defence against reactive oxygen metabolites. The authors suggest *SC* may act by either directly scavenging the reactive oxygen metabolites, due to the presence of various antioxidant compounds, or by increasing the synthesis of antioxidant molecules. These findings raise the question of the extent to which fruit skin extract may exhibit antidiabetic properties in the case of diabetes, given that it contains higher levels of bioactive compounds and demonstrates stronger antioxidant activity than seed extract. Therefore, further studies in this area are strongly warranted.

**Table 3:** Summary of bioactive compounds in bitter melon (Momordica charantia), jambolan plum (Syzygium cumini), and gurmar (Gymnema sylvestre): Mechanisms of action, plant sources, and specific compounds with potential antidiabetic activity.

Group of Active Compounds	Key Biological Actions	Plant	Representative Compounds with Reported Antidiabetic Potential
**Polyphenols** **Flavonoids** **Tannins**	↓ ROS and oxidative stress ↓ lipid peroxidation ↑ GSH levels ↑ antioxidant enzymes (SOD, CAT, GPx, HO-1, NQO1) ↑ Nrf2/ARE signalling ↓ NF-κB activation ↓ pro-inflammatory cytokines (TNF-α, IL-1β, IL-6) ↓ COX-2 and iNOS expression ↓ apoptosis/cellular damage ↑ insulin sensitivity ↑ glucose uptake ↓ blood glucose levels [148,149] ↑ AMPK activation [150] ↓ G6Pase activity [151] ↓ α-amylase activity [152] ↓ α-glucosidase activity [153] ↓ intestinal glucose absorption [154]	Bitter melon Gurmar Jambolan Bitter melon Gurmar Jambolan Gurmar Jambolan	Gallic acid [155] Gallic acid [155] Quercetin [89,151,156] Vanillin [89] Quercetin [151,156] Kaempferol [157] Vanillin [158] Rutin [159]
**Triterpenoids/** **Saponins**	↓ ROS and oxidative stress ↓ lipid peroxidation ↑ GSH levels ↑ antioxidant enzymes (SOD, CAT, GPx, GSH) ↑ Nrf2/ARE signalling ↑ AMPK activity ↑ AMPK/PI3K signalling ↑ Akt phosphorylation (p-Akt) ↑ insulin signalling (IR/IRS/PI3K/Akt) ↑ insulin sensitivity ↑ glucose uptake ↓ blood glucose levels ↓ NF-κB activation ↓ pro-inflammatory cytokines (TNF-α, IL-6) ↓ COX-2 and iNOS expression ↓ cellular damage [160,161,162]	Bitter melon Gurmar	Momorcharaside A [163] Momordicoside A [163] Karaviloside XI [160,163] Curcumin [164] Gymnemic acid [104,160] Lupeol [165]
**Polysacharides**	↓ ROS levels ↓ oxidative stress ↓ lipid peroxidation ↑ GSH levels ↑ antioxidant enzymes (SOD, CAT, GPx) ↑ Nrf2/ARE signalling ↓ NF-κB activation ↓ pro-inflammatory cytokines (TNF-α, IL-6, IL-1β) ↑ AMPK signalling ↑ insulin sensitivity ↑ glucose uptake ↓ blood glucose levels ↑ gut barrier integrity ↑ beneficial gut microbiota [162,166,167,168]	Bitter melon	Galactose [169] Rhamnose [169] Arabinose [169] Xylose [169]
**Proteins/peptides**	↓ H_2_O_2_ levels ↓ ROS levels ↓ oxidative stress ↓ lipid peroxidation ↑ antioxidant defence ↑ redox homeostasis [170,171]	Bitter melon Gurmar	Polypeptide-P (p-insulin) [172] mcIRBP/BG68 [173,174] Polypeptide-k [175] Charantin [100] Gurmarin [102]

To make this paper easier to follow, we present the active compounds and their action summarised in the table. Modify from the works presented in table, in square brackets. Abbreviations—see the list of Abbreviations at the end of the paper. (↑) enhancement of action, (↓) lowering of action.

## 5. Conclusions and Prospects

The 21st century is experiencing an unprecedented epidemic of metabolic diseases, with DM at the forefront of them, being the driving force in this crisis. DM causes severe complications, incriminating for the patient, both physically and emotionally, for society—financially. This is due to, among other things, severe complications including CVD, blindness (retinopathy), kidney failure, and neuropathy leading to foot amputations.

With over 537 million adults now living with diabetes—a figure projected to rise to over 780 million by 2045—the disease has become one of the most significant global health challenges [176]. Therefore, it is important to prevent early onset and development of insulin resistance, hyperglycaemia and stabilisation of oxidative balance, by early initiation of modification of the daily diet, inclusion of foods with strong antioxidant properties or stabilizing the oxidative balance in the organism.

This review presents, that antioxidant antidiabetic action mostly mentioned three plans are mediated through multiple mechanisms: enhancing insulin secretion, improving insulin secretion, and improving insulin sensitivity.

The available evidence suggests that plants used for centuries in traditional medicinal systems may have considerable potential for regulating carbohydrate metabolism, at least in part through their antioxidant properties. These effects appear to play an important role in both the prevention and management of diabetes, primarily by protecting pancreatic cells and reducing chronic inflammation.

Ayurvedic medicine and plant-oriented medicine are most appropriately used as adjunctive therapy rather than as a replacement for conventional drugs.

Despite these promising findings, several important limitations must be acknowledged. Existing studies differ substantially in terms of plant origin, geographical cultivation conditions, and plant parts used for extract preparation. Moreover, extraction procedures vary considerably among studies. This issue is particularly significant because the choice of solvent directly affects the extraction efficiency of bioactive compounds and, consequently, their biological activity.

Although plant-derived drugs show promise for managing prediabetes or acting as complementary therapies, they require rigorous long-term preclinical and clinical studies, as well as increased safety monitoring and standardisation of production. Further in vivo studies and clinical trials are needed to safely use these natural resources for human health. Specifically, future research should investigate factors such as dose concentration and toxicology.

Collectively, these factors limit the comparability and reproducibility of current findings, making it difficult to establish reliable evidence for the clinical application of these plant-derived preparations. Nevertheless, comprehensive review studies that summarise the currently available data constitute an important step toward improving our understanding of their therapeutic potential and may contribute to the development of more standardised and detailed future research.

## Figures and Tables

**Table 1:** Biological action of **prooxidant** enzymes directly involved in the ROS generation system.

No.	Enzyme Name	Biological Action Description
**1.**	NADPH Oxidase (NOX)	These widespread enzymes use NADPH to turn oxygen into superoxide in almost all human tissues. The cytosolic membrane binds seven distinct oxidase isoforms with varying tissue distributions, while NADPH concentration dictates the cellular redox balance. NADPH generation relies on glucose metabolism, a pathway critical for broader cellular redox balance independent of direct ROS action. In diabetic rats, elevated ROS stress triggers an upregulation of the pentose phosphate pathway, which restores NADPH reserves.
**2.**	Uncoupled Nitric Oxide Synthase (NOS)	NO, a crucial gas for maintaining vascular homeostasis, is synthesised through arginine oxidation by three distinct NOS enzymes. By regulating blood pressure and platelet aggregation, NO functions as a key vasodilator within the cardiovascular system. Hyperglycaemia causes a deficit in the cofactor of NO synthesis, as tetrahydrobiopterin (BH4) is oxidised to 7,8-dihydrobiopterin (BH2). Then, the eNOS dimer splits into a monomer and loses its normal function. It starts making ROS, including superoxide. Finally, superoxide reacts fast with NO to form peroxynitrite.
**3.**	Cyclooxygenase (COX/ProstaglandinG/H Synthase (PGHS)	Comprising two to three distinct enzymes, this family governs the biosynthesis of pro-inflammatory eicosanoids, including prostacyclin, prostaglandins, and thromboxane. By catalysing the rate-limiting step, these enzymes bridge OS and inflammatory pathways. The catalytic process utilises molecular oxygen to generate (endo)peroxidase intermediates alongside ROS byproducts.
**4.**	Lipooxygenase (LPOs)	Functioning similarly to PGHS, this diverse group of iron-dependent oxidases drives inflammatory cascades and reactive oxygen species (ROS) generation. They facilitate the peroxidation of membrane-bound unsaturated fatty acids, producing oxygenated intermediates that mature into signalling mediators such as leukotrienes and lipoxins, alongside parallel ROS by-production. Depending on the specific isoform—of which there are up to 13—these enzymes exhibit distinct substrate preferences and site-specific oxygenation patterns.
**5.**	Xantine Oxidase (XO)	Similar to eNOS, XO exhibits ROS-free dehydrogenase activity under normal physiological states. However, oxidative stress triggers a conformational shift from its reductase to its oxidase form. This new form reduces molecular oxygen to superoxide and H_2_O_2_, thereby amplifying OS in endothelial, epithelial, and polymorphonuclear cells, as well as fibroblasts. This mechanism worsens the renal and vascular impairments often seen in diabetes.
**6.**	Heme Oxidase (HO-1)	The heme degradation pathway yields both pro-oxidant and antioxidant agents, thereby modulating cellular redox status. Because Nrf2 regulates HO-1 induction, this enzyme functions as an essential component of the cellular antioxidant defence against OS. This protective role stems from the antioxidant capacity of downstream metabolites biliverdin and bilirubin. Although peak HO-1 expression occurs in the spleen, its role in endothelial cells is vital. Upregulating this pathway enhances insulin secretion and mitigates hyperglycaemia severity.
**7.**	Myeloperoxidase (MPO)	This enzyme exhibits a specific redox potential relative to other peroxidases. It utilises hydrogen peroxide to oxidise chloride into hypochlorite, an oxidant species associated with ROS linked to the degradation of microbial pathogen components. MPO activity levels differ in diabetic neutrophils, where neutrophil phagocytosis and killing functions show altered metrics. Additionally, the enzyme participates in the oxygenation of serum lipoproteins and endothelial cell components, concurrent with a reduction in nitric oxide and decreased cell viability during hyperglycaemia.
**8.**	Cytochrome P-450 (CYP)	These haemoprotein monooxidases—found in mitochondrial and microsomal fractions—metabolise endogenous bioactive molecules (such as bile acids, corticoids, cholecalciferol and prostaglandins) and xenobiotics (like drugs and toxins). Their catalytic activity primarily generates superoxide alongside other ROS. Hyperglycaemia strongly induces the CYP2E1 mitochondrial isoform.

**Table 2:** Biological action of **antioxidant** enzymes and antioxidant related proteins.

No.	Enzyme Name	Biological Action Description
**1.**	Superoxide Dismutase (SOD)	SOD enzymes catalyse the dismutation of superoxide into H_2_O_2_. Because H_2_O_2_ is released as the final product of this enzymatic reaction, SOD does not entirely eliminate ROS signalling. Under pathological conditions characterised by electron transport chain (ETC) impairment—such as diabetes and related metabolic disorders—an excessive accumulation of mitochondrial superoxide is accompanied by a significant reduction in mitochondrial SOD activity.
**2.**	Catalases and Peroxidases (CAT and PO)	Both enzyme families utilise H_2_O_2_ as a substrate. Current evidence indicates that CAT activity plays a minor role in the primary antioxidant response, given that the expression of its encoding gene is not regulated by the Nrf2 pathway. Furthermore, in mammals, CAT is localised exclusively within the peroxisomes of specific tissues, predominantly the kidneys, liver and lungs
**3.**	Peroxiredoxins (Prx)	Prx constitute a family of mammalian antioxidant enzymes that facilitate H_2_O_2_. scavenging. Although functionally analogous to CAT and peroxidases (PO), Prx operates via a distinct catalytic mechanism and mediates antioxidant and anti-inflammatory pathways across diverse cell types. While mammalian systems express up to six Prx isoforms, these are classified into three primary structural groups based on oligomeric state and specific cofactor requirements.
**4.**	Thioredoxin (Trx) and Related Proteins (Trx Reductase and TxNPI)	Thioredoxins function as key thiol-oxidoreductases that principally regulate thiol-sensing pathways within cellular redox signalling networks. Synthesised via the Nrf2-ARE signalling pathway, this family of low-molecular-weight proteins maintains cellular homeostasis by reducing both disulfide bridges and sulfenic acids back to their native cysteine (Cys) residues. This reductive activity targets numerous intracellular substrates, including essential antioxidant enzymes such as Prx. Similar to SOD isoforms, Prx variants are compartmentalised within specific subcellular niches. Both enzyme families are predominantly localised within the cytosol and the mitochondria.
**5.**	Paraoxonases (PON)	PON are plasma proteins bound to HDL that possess esterase and oxidoreductase activities. They protect blood lipoproteins from lipid oxidation, particularly LDL, and help maintain plasma redox balance to lower cardiovascular risk, even though they do not neutralise ROS directly. Because their activity drops in hyperglycaemia and aging, PON levels serve as a clinically important biochemical marker for blood redox status.
**6.**	Sirtuins (Sirt)	This family of proteins are NADPH^+^- dependent protein deacetylases. Currently, this seven-member family is split across different parts of the cell: Sirt1 and Sirt2 move between the cytoplasm and nucleus, Sirt3 sits in the mitochondrial matrix, and Sirt6 stays strictly in the nucleus. Sirt help extend lifespan (an antiaging action) and may play a key role in the development of T2D. Multiple studies indicate that Sirt expression is reduced in diabetic states. Conversely, diminished Sirt levels promote oxidative stress, impair glucose metabolism, and accelerate diabetic complications. This highlights the complex interplay between hyperglycaemia and OS.

## Data Availability

No new data were created or analysed in this study. Data sharing is not applicable to this article.

## Abbreviations

The list of abbreviations used in this manuscript:

ABTS	2,2′-Azino-bis(3-ethylbenzothiazoline-6-sulfonic acid)
ABTS+	ABTS radical cation
ADP	Adenosine diphosphate
AGE	Advanced glycation end-product
AGE–RAGE	Advanced glycation end-product–receptor for advanced glycation end-products
Akt	Protein kinase B
ARE	Antioxidant response element
ATP	Adenosine triphosphate
BH4	Tetrahydrobiopterin
CAT	Catalases
Ca^2+^	Calcium ion
COX	Cyclooxygenase
COX-2	Cyclooxygenase-2
CTLA-4	cytotoxic T-lymphocyte-associated protein 4
CYP	Cytochrome
DKA	Diabetic ketoacidosis
DM	Diabetes mellitus
DNA	Deoxyribonucleic acid
ED	Endothelial dysfunction
eNOS	Endothelial nitric oxide synthase
ERK	Extracellular signal-regulated kinase
ET-1	Endothelin-1
ETC	Electron transport chain
FADH_2_	Flavin adenine dinucleotide (reduced form)
G6P	Glucose-6-phosphate
GDM	Gestational diabetes mellitus
GFAT	Glutamine-fructose-6-phosphate amidotransferaseGrb
GLUT4	Glucose transporter type 4
GPx	Glutathione peroxidase
Grb	Growth factor receptor-bound protein
GS	*Gymnema sylvestre* (L.) Skeels
GSH	Reduced glutathione
GSK3β	Glycogen synthase kinase 3 beta
HbA1c	Haemoglobin A1c
HDL	High-density lipoprotein
Hes1	Hairy and enhancer of split-1
HHS	Hyperosmolar hyperglycaemic state
HLA	Human leukocyte antigen
HO-1	Heme oxygenase-1
HOMA-IR	Homeostatic Model Assessment of Insulin Resistance
H_2_O_2_	Hydrogen peroxide
IC50	Half maximal inhibitory concentration
IKK-β	inhibitor of nuclear factor kappa B kinase (subunit) beta
IL-6	Interleukin-6
iNOS	Inducible nitric oxide synthase
IR	Insulin resistance
JNK	c-Jun N-terminal kinase
KATP channel	ATP-sensitive potassium channel
Keap1	Kelch-like ECH-associated protein 1
LDL	Low-density lipoprotein
LPO	Lipooxygenase
LPS	Lipopolysaccharide
MAPK	Mitogen-activated protein kinase
MC	*Momordica charantia* L.
MCPs	*Momordica charantia* polysaccharides
MIN6	Mouse insulinoma 6 cells
MODY	Maturity-onset diabetes of the young
MPO	Myeloperoxidase
mRNA	Messenger ribonucleic acid
mtDNA	Mitochondrial DNA
NADPH	Nicotinamide adenine dinucleotide phosphate
NF-κB	Nuclear factor kappa B
nNOS	Neuronal nitric oxide synthase
NO	Nitric oxide
NOS	Nitric oxide synthase
NOX	NADPH oxidase
Nrf2	Nuclear factor erythroid 2–related factor 2
OSA^®^	Aqueous alcohol extract of fresh *Gymnema sylvestre*
ORAC	Oxygen radical absorbance capacity
OS	Oxidative stress
O-GlcNAcylation	O-linked N-acetylglucosaminylation
PAI-1	Plasminogen activator inhibitor-1
PD-1	Programmed cell death protein 1
Pdx1	Pancreatic and duodenal homeobox 1
PGHS	Prostaglandin G/H synthase
PI3K	Phosphoinositide 3-kinase
PKC	Protein kinase C
PON	Paraoxonase
PO	Peroxidase
Prx	Peroxiredoxin
RAGE	Receptor for advanced glycation end-products
RIPs	Ribosome-inactivating proteins
RONS	Reactive oxygen and nitrogen species
ROS	Reactive oxygen species
SC	*Syzygium cumini*
Shc	Src homology and collagen protein
SOD	Superoxide dismutase
STZ	Streptozotocin
T1DM	Type 1 diabetes mellitus
T2DM	Type 2 diabetes mellitus
TNF-α	Tumour necrosis factor alpha
VEGF	Vascular endothelial growth factor
VSM	Vascular smooth muscle
Wnt	Wingless-related integration site signalling pathway
